# The relationship between sex hormone levels and ocular surface parameters in girls with idiopathic central precocious puberty

**DOI:** 10.3389/fendo.2024.1429154

**Published:** 2024-07-25

**Authors:** Wen Jiang, Lixia Yang, Shuang Liang

**Affiliations:** ^1^ Institute of Medical Sciences, The Second Hospital of Shandong University, Shandong, China; ^2^ Department of Ophthalmology, The Second Hospital of Shandong University, Shandong, China; ^3^ Department of Pediatrics, The Second Hospital of Shandong University, Shandong, China

**Keywords:** sex hormone, children, ocular surface, central precocious puberty, objective scatter index, dry eye disease

## Abstract

**Purpose:**

The study aimed to investigate the correlation between the change of sex hormone levels and ocular surface parameters in girls with idiopathic central precocious puberty(ICPP).

**Methods:**

Eighteen girls with ICPP and 18 age-matched normal girls participated in this study, all of the participants had undergone physical measurements, laboratory tests, imaging examination and ocular surface assessments.

**Results:**

The Objective Scatter Index (OSI) in the ICPP group was significantly higher than in the control group (*P* = 0.031), girls with ICPP showed slightly lower MNITBUT compared to the normal control group, although this difference was not statistically significant. Bivariate analysis revealed a positive association between estradiol and OSI (r=0.383, *P*=0.021), Additionally, in the study population, both Luteinizing hormone (LH) and Follicle-stimulating hormone (FSH) were negatively correlated with Mean noninvasive tear breakup time (MNITBUT) (r=-0.359, *P*=0.031)(r=-0.357, *P*=0.032).

**Conclusion:**

In comparison with the normal control group, alterations in the OSI were observed in girls with ICPP. This alteration may be associated with an elevation in estrogen levels. Although there was a slight non-significant decrease in NITBUT in ICPP girls, the negative correlation between LH and FSH with MNITBUT suggests new perspective for further investigation.

## 1 Introduction

Dry eye disease (DED) is a multifactorial condition characterized by inflammation and loss of tear membrane homeostasis (1). Symptoms such as dryness, grittiness, burning sensation, and fluctuating vision can reduce the quality of life and impact work efficiency and sleep quality, significantly affecting personal health (2–4). Currently, the global incidence of dry eye is estimated to range from 5% to 50% (5). Although DED is known to affect all ages, there is limited literature on pediatric DED or ocular surface, likely due to the limited subjective expression in children and differing clinical manifestations compared to adults, leading to its diagnosis being often overlooked. However, in recent years, the incidence of dry eyes has been becoming younger, and the incidence of dry eyes in children and adolescents has been increasing (6). According to the data analysis of the US health care system, the prevalence of dry eyes in people aged 2 to 17 years was 0.20% from 2003 to 2015 (7). Compared to other countries, the reported prevalence of pediatric dry eye in China is relatively higher (8). Therefore, it is crucial to focus on the occurrence of pediatric dry eye and changes in ocular surface parameters.

Sex Hormones are associated with the physiology and pathophysiology of almost all organs as well as most diseases, and ocular surface tissues might act as targets for sex hormones, might experience local effects of these sex hormone (9, 10). In recent years, there has been an increasing interests in the relationship between sex hormones and ocular surface tissues. Currently, most studies have shown a protective effect of androgens on the ocular surface (11–13). Androgens play a nutritional role in the growth and function of meibomian gland (14), while also exerting the anti-inflammatory effects (15). Additionally, Clinical studies confirmed that lack of androgens might contribute to a higher incidence of dry eye (16, 17). However, the role of estrogen in dry eye is still unclear, which is a highly controversial topic. Animal evidence and human *in vitro* studies have shown that estrogen could inhibit the secretion of lacrimal gland (12) and might promote DED by upregulating the NLRP 3 inflammasome (18). However, there are also reports showing that estrogen also has an anti-inflammatory effect on the ocular surface (19). Clinical studies have also emerged with conflicting results. High estrogen levels could worsen dry eye symptoms (20), while other evidence suggests that estrogen reduction is associated with decreased tear function and increased risk of DED (21–23). Additionally, the impact of estrogen replacement therapy on ocular surface in postmenopausal women remains controversial (24, 25). A better understanding of the effects of sex hormones on the ocular surface is important for further prevention and control of DED. Therefore, we should pay attention to changes in ocular parameters in populations with hormonal fluctuations.

In children, the incidence of precocious puberty in women increases year by year, which has attracted wide attention from pediatric endocrinologists (26, 27). Whether the premature initiation of the hypothalamic and pituitary gonadal axis (HPGA) and the consequent changes in sex hormones would induce changes in ocular surface parameters is the current research gap. Therefore, this study aims to clarify whether girls with idiopathic central precocious puberty (ICPP) exhibit changes in ocular surface parameters and to establish the correlation between sex hormones and ocular surface parameters.

## 2 Materials and methods

### 2.1 Subjects

This study was conducted from December 2020 to December 2023 at the Second Hospital of Shandong University. All the subjects included in this study were children who sought medical attention for the first time due to precocious puberty and were diagnosed with ICPP. During the study period, none of the subjects received any drug interventions, including gonadotropin-releasing hormone analogs. The diagnostic criteria for ICPP were based on the guidelines referenced from the Chinese Medical Association (28): (1) Onset of secondary sexual characteristics in girls before age 8 years or menarche occurring under age 10 years; (2) Accelerated linear growth, bone age(BA) exceeding chronological age by 1 year or more; (3) Enlargement of the gonads: pelvic ultrasound shows increased uterine and ovarian volume in girls, with multiple ovarian follicles >4 mm in diameter; (4) Activation of the HPGA: The serum levels of gonadotropins and sex hormones reached pubertal levels. Exclusion criteria comprised: (1) Secondary central precocious puberty with a clearly defined organic cause; (2)Participants taking known medications affecting the HPGA or having used steroid medications prior to the study; (3) Peripheral precocious puberty; (4) Known endocrine diseases or chromosomal abnormalities were omitted from the study; (5) Severe organic diseases and incomplete medical records; (6) Individuals with corneal inflammation, blepharitis, conjunctival stones, entropion, and abnormal blinking due to local eye diseases and ocular irritation factors were excluded; (7) Those with abnormal blinking resulting from refractive errors, drugs, psychological, and systemic illnesses were also excluded; (8) Individuals with a history of eye trauma, surgery, or corneal contact lens wear were not included.

PASS 11 software was used to calculate the sample size based on the tear breakup time (TBUT) in polycystic ovary syndrome women and controls in previous study (29). For 90% power at alpha = 0.05, a minimum of 13 participants were required to complete the study. Ultimately, a total of 18 girls with ICPP and 18 age-matched normal girls participated in this study. The research protocol received approval from the Institutional Review Board of the hospital, adhering to the principles of the Helsinki Declaration. Prior to the study commencement, written informed consent was obtained from both the children and their parents for their participation in the study.

### 2.2 Anthropomorphic measurements

The physical examinations were conducted by the same pediatric endocrinologist who had received professional training. All participants underwent measurements of height, weight and assessment of sexual development. Body weight was measured using a standard electronic scale with a precision of 0.1 kg, and height was assessed using a standard height stadiometer (TJ-220S-18, Beideneng, Shanghai, China) with a precision of 0.1 cm. Pubertal stage was evaluated based on the Tanner criteria (30). Body Mass Index (BMI) was calculated as weight divided by height squared and expressed in kg/m2.

### 2.3 Laboratory assessments

All participants underwent fasting blood tests to assess endocrine hormone levels, liver and kidney function, and metabolic markers. Free triiodothyronine (FT3), Free thyroxine (FT4), Thyroid-stimulating hormone (TSH), Luteinizing hormone (LH), Follicle-stimulating hormone (FSH), Estradiol, Testosterone, Progesterone, Prolactin were quantified using a chemiluminescence analyzer (DXI800, Beckman Coulter, USA). Adrenal corticotropic hormone (ACTH) and Cortisol (COR) were analyzed using an auto biochemical analyzer (AutoLumoA2000Plus, Antu biology, Zhenzhou, China). IGF-1 were quantified using automatic luminescence tester (Malumi X8, New industry, Shenzhen, China). Total cholesterol (TC), High-density lipoprotein cholesterol (HDL-C), Low-density lipoprotein cholesterol (LDL-C), Triglycerides (TG), Fasting plasma glucose (FPG), C-reactive protein (CRP), Uric acid, Alanine aminotransferase (ALT), and blood urea nitrogen (BUN) were analyzed using an automatic biochemical analyzer (CobasC702, Roche Diagnostics, Shanghai, China). Fasting insulin levels were measured using fully automatic chemiluminescence analyzer (CobasE602, Roche Diagnostics, Shanghai, China). Insulin resistance was assessed using the homeostasis model assessment index (HOMA-IR), calculated as fasting insulin multiplied by fasting glucose, divided by 22.5 (31).

All premenarchal participants in the ICPP group required completion of the gonadotropin-releasing hormone (GnRH) stimulation test as part of their clinical assessment. Baseline blood samples were collected before intravenous injection of gonadotropins (2.5 μg/kg, maximum 0.1 mg), and LH and FSH were collected at 30, 60, and 90 minutes after the injection. The GnRH stimulation test indicated that a peak LH level >5 mIU/mL and an LH/FSH ratio >0.6 suggested activation of the gonadal axis.

### 2.4 Imaging parameters

All participants underwent bone age assessment using radiographs of the left hand and wrist following the Greulich and Pyle method. The bone age-chronological age (BA-CA) was then calculated. In addition, all girls with ICPP underwent hypothalamic-pituitary MRI and gynecological ultrasound. The MRI was conducted using a 3.0T scanner (Siemens, Erlangen, Germany) with a slice thickness of 3mm. Gynecological ultrasound was performed using a 3.5-5 MHz mechanical sector scanner (GE Healthcare, Tiefenbach, Austria). Measurements of the entire uterus, body and cervix, as well as the longitudinal, anteroposterior, and transverse diameters of the ovaries. Follicle count and size were observed and recorded.

### 2.5 Ocular surface parameters

All of the participants completed the ophthalmic examination, and data used in this study were obtained from the right eye of each subject. The Lipid Layer Thickness (LLT) was measured, and the incomplete blinking behavior of the subjects was recorded using a LipiView ocular interferometer. Additionally, the participant’s partial blink rate (PBR) was calculated. The Tear Meniscus Height (TMH) was measured using a non-invasive Tear Film Analysis System (Tearscope Plus, Keeler, Germany). Non-invasive Tear Break-Up Time (NITBUT) and Mean Non-invasive Tear Break-Up Time (MNITBUT) measurements were conducted using an ocular surface integrated analysis instrument (Daecometer, USA). FNITBUT represented the result of the first measurement, while MNITBUT was calculated as the average of three consecutive measurements. The OSI and Mean Ocular Surface Interferometry (MOSI) were measured using The Optical Quality Analysis System (OQAS, Visiometrics, Terrassa, Spain). All measurements were performed by the same ophthalmic technician.

### 2.6 Statistical analysis

The Statistical Package for the Social Sciences (SPSS) version 20.0 (SPSS Inc., Chicago, USA) was used for the statistical analysis. Normally distributed variables were reported as mean (SD), while skewed distributed variables were presented as median (interquartile range). Categorical variables were compared by the chi square test. Group comparisons were conducted using Students’ t-tests or Mann-Whitney U tests. The correlation between the variables was assessed using Spearman correlation analysis. Statistical significance was considered for differences with a p-value of less than 0.05.

## 3 Results

### 3.1 Baseline characteristics of study subjects

The characteristics of the anthropometric and metabolic markers in the study subjects are presented in **Table 1**. The age of ICPP group and the control group was matched (8.15 ± 1.45 *vs.* 7.77 ± 1.24, *P*=0.902). There were no statistically significant differences in ALT and BUN, as well as FBG and lipid metabolism between the two groups (all *P*>0.05). However, the ICPP group exhibited significantly higher levels of BMI, insulin, and HOMA-IR compared to the control group(all *P*<0.001).

**Table 1:** Clinical and laboratory characteristics of study participants.

Variable	ICPP group (n = 18)	Control group (n =18)	*P* value
Age (yr)	8.15 (1.45)	7.77 (1.24)	0.902
BMI (kg/m2)	18.79 (2.99)	15.83 (1.71)	<0.001*
CRP (mg/L)	1.31 (0.10-3.05)	0.94 (0.21-1.34)	0.851
Insulin (uU/mL)	15.02 (8.31-22.83)	6.62 (4.37-8.71)	<0.001*
FBG (mmol/L)	5.00 (0.48)	4.73 (0.40)	0.077
HOMA-IR	3.29 (1.80-5.37)	1.44 (0.77-1.90)	<0.001*
ALT (U/L)	15.72 (6.65)	15.50 (5.67)	0.915
BUN (mmol/L)	3.91 (0.84)	3.87 (0.84)	0.880
uric acid (μmol/L)	291.62 (63.62)	262.47 (52.65)	0.144
TC (mmol/L)	3.88 (0.62)	4.23 (0.96)	0.194
HDL-C (mmol/L)	1.36 (1.03-1.51)	1.33 (1.29-1.46)	0.214
LDL-C (mmol/L)	2.19 (0.60)	2.42 (0.85)	0.344
TG (mmol/L)	0.69 (0.63-0.86)	0.68 (0.58-0.78)	0.443

BMI, body mass index; CRP, C-reactive protein; FBG, Fasting blood glucose; HOMA-IR, homeostasis model assessment of IR; ALT, Alanine aminotransferase; BUN, Blood urea nitrogen; TC, total cholesterol; TG, triglycerides; HDL-C, high-density lipoprotein cholesterol; LDL-C, low-density lipoprotein cholesterol; ALT, alanine aminotransferase.

*P<0.05.

The endocrine parameters in the ICPP and control groups are presented in **Table 2**. As expected, the ICPP group exhibited significantly higher levels of LH, FSH, estradiol, testosterone, IGF-1 and BA-CA compared to the normal control group. The levels of progesterone, prolactin, FT3, FT4, TSH, ACTH and COR were similar in both groups (all *P* > 0.05).

**Table 2:** Endocrine factors in in ICPP and Control groups.

Variable	ICPP group (n=18)	Control group (n=18)	*P* value
IGF-1 (ng/mL)	321.78 (110.91)	168.17 (44.57)	<0.001*
BA-CA (yr)	1.85 (1.00-3.00)	0.00 (-0.15-0.00)	<0.001*
FT3 (pmmol/L)	6.45 (0.72)	6.55 (0.96)	0.716
FT4 (pmmol/L)	11.56 (10.64-13.95)	11.82 (11.37-12.85)	0.584
TSH (μU/mL)	3.36 (1.72-4.33)	2.64 (2.14-5.10)	0.673
ACTH (mmol/L)	24.55 (15.18-33.82)	23.89 (17.62-29.94)	0.584
COR (pg/mL)	273.42 (124.22)	280.25 (127.80)	0.872
LH (mIU/mL)	2.55 (0.83-3.98)	0.22 (0.10-0.47)	<0.001*
FSH (mIU/mL)	6.06 (2.73)	2.36 (1.40)	<0.001*
Estradiol (pg/ml)	26.00 (16.41-54.53)	15.00 (6.81-16.00)	<0.001*
Testosterone (ng/ml)	0.20 (0.04-0.32)	0.10 (0.20-0.19)	0.019*
Progesterone (ng/ml)	0.44 (0.23-0.67)	0.31 (0.19-0.60)	0.126
Prolactin (ng/ml)	8.74 (5.49-17.27)	9.84 (7.16-12.10)	0.767

IGF-1, Insulin-like growth factor 1; BA-CA,Bone age-Chronological age;FT3, Free triiodothyronine; FT4, Free thyroxine; TSH, Thyroid-stimulating hormone; ACTH, Adrenal corticotropic hormone; COR,Cortisol; LH, Luteinizing hormone; FSH, Follicle-stimulating hormone.

*P<0.05.

**Table 3** displays the ocular surface parameters of the study population. The OSI in the ICPP group was significantly higher than in the control group(*P* = 0.031), girls with ICPP showed slightly lower MNITBUT compared to the normal control group, although this difference was not statistically significant. In addition, LLT, FNITBUT, TMH, PBR and MOSI were similar between the two groups (all *P* > 0.05).

**Table 3:** Ocular surface parameters in ICPP and Control groups.

Variable	ICPP group (n = 18)	Control group (n =18)	*P* value
LLT (nm)	67.44 (27.74)	61.94 (29.08)	0.565
FNITBUT (s)	4.92 (3.24-8.99)	4.07 (3.06-9.05)	0.864
MNITBUT (s)	7.19 (3.55)	8.60 (4.33)	0.292
TMH (mm)	0.11 (0.09-0.13)	0.11 (0.10-0.12)	0.628
PBR (%)	31.50 (1.00-81.50)	46.00 (1.00-80.50)	0.179
MOSI	0.99 (0.67-2.99)	1.17 (0.52-1.93)	0.696
OSI	0.45 (0.30-0.90)	0.25 (0.20-0.53)	0.031*

LLT, Lipid layer test; FNITBUT, First noninvasive tear breakup time; MNITBUT, Mean noninvasive tear breakup time; TMH, Tear meniscus height; PBR, Partial blinks rate; MOSI, Mean objective scatter index; OSI, Objective scatter index.

*P<0.05.

### 3.2 Correlations between LH and parameters of ocular surface

We performed a bivariate correlation analysis to explicit the relationship between LH and ocular surface parameters in study subjects are shown in **Table 4**. LH was significantly negatively associated with MNITBUT(r=-0.359, *P*=0.031), while no association was found between LH and other ocular surface parameters (all *P* > 0.05).

**Table 4:** Correlation between LH and parameters of Ocular surface.

Variable	r	*P*
LLT (nm)	0.084	0.627
FNITBUT (s)	-0.169	0.326
MNITBUT (s)	-0.359	0.031*
TMH (mm)	0.168	0.328
PBR (%)	-0.052	0.764
MOSI	-0.105	0.544
OSI	0.291	0.085

LLT, Lipid layer test; FNITBUT, First noninvasive tear breakup time; MNITBUT, Mean noninvasive tear breakup time; TMH, Tear meniscus height; PBR, Partial blinks rate; MOSI, Mean objective scatter index; OSI, Objective scatter index.

*P<0.05.

### 3.3 Correlations between FSH and parameters of ocular surface

**Table 5** displays the correlations between FSH and ocular surface parameters in the study subjects. Similar to LH, FSH showed a negative correlation with MNITBUT (r=-0.357, P=0.032). However, no significant associations were found between FSH and other ocular surface parameters (all *P* > 0.05).

**Table 5:** Correlation between FSH and parameters of Ocular surface.

Variable	r	*P*
LLT (nm)	0.246	0.149
FNITBUT (s)	-0.211	0.217
MNITBUT (s)	-0.357	0.032*
TMH (mm)	0.060	0.726
PBR (%)	0.141	0.412
MOSI	-0.268	0.114
OSI	0.169	0.325

LLT, Lipid layer test; FNITBUT, First noninvasive tear breakup time; MNITBUT, Mean noninvasive tear breakup time; TMH, Tear meniscus height; PBR, Partial blinks rate; MOSI, Mean objective scatter index; OSI, Objective scatter index.

*P<0.05.

### 3.4 Correlations between estradiol and parameters of ocular surface

The bivariate correlation analysis in **Table 6** showed a significant positive association between estradiol and OSI (r=0.383, *P*=0.021), indicating a positive correlation between estradiol and OSI. However, no significant associations were found between estradiol and other ocular surface parameters (all *P* > 0.05).

**Table 6:** Correlation between Estradiol and parameters of Ocular surface.

Variable	r	*P*
LLT (nm)	-0.091	0.598
FNITBUT (s)	-0.207	0.226
MNITBUT (s)	-0.258	0.129
TMH (mm)	0.129	0.453
PBR (%)	0.041	0.812
MOSI	0.046	0.792
OSI	0.383	0.021*

LLT, Lipid layer test; FNITBUT, First noninvasive tear breakup time; MNITBUT, Mean noninvasive tear breakup time; TMH, Tear meniscus height; PBR, Partial blinks rate; MOSI, Mean objective scatter index; OSI, Objective scatter index.

*P<0.05.

### 3.5 Correlations between testosterone and parameters of ocular surface

**Table 7** shows that there is no significant correlation between testosterone and the indicators of ocular surface in the study population(all *P* > 0.05).

**Table 7:** Correlation between Testosterone and parameters of Ocular surface.

Variable	r	*P*
LLT (nm)	-0.055	0.749
FNITBUT (s)	0.283	0.095
MNITBUT (s)	0.112	0.517
TMH (mm)	0.194	0.258
PBR (%)	-0.101	0.557
MOSI	-0.074	0.668
OSI	0.133	0.440

LLT, Lipid layer test; FNITBUT, First noninvasive tear breakup time; MNITBUT, Mean noninvasive tear breakup time; TMH, Tear meniscus height; PBR, Partial blinks rate; MOSI, Mean objective scatter index; OSI, Objective scatter index.

### 3.6 Correlations between progesterone and parameters of ocular surface

In the study population, there is no statistically significant correlation observed between progesterone and the indicators of the ocular surface (all *P* > 0.05, see **Table 8**).

**Table 8:** Correlation between Progesterone and parameters of Ocular surface.

Variable	r	*P*
LLT(nm)	0.06	0.727
FNITBUT (s)	-0.193	0.261
MNITBUT (s)	-0.311	0.065
TMH(mm)	0.323	0.055
PBR (%)	-0.101	0.557
MOSI	-0.037	0.828
OSI	0.022	0.898

LLT, Lipid layer test; FNITBUT, First noninvasive tear breakup time; MNITBUT, Mean noninvasive tear breakup time; TMH, Tear meniscus height; PBR, Partial blinks rate; MOSI, Mean objective scatter index; OSI, Objective scatter index.

## 4 Discussion

To best of our knowledge, this study is the first attempt to report changes in ocular surface parameters and their correlation with sex hormones in girls with ICPP. In this study, we found that the OSI was significantly higher in the ICPP group than in the control group. In addition, there was a positive correlation between estradiol and OSI, Additionally, in the study population, both LH and FSH were negatively correlated with MNITBUT.

In this research, girls with ICPP demonstrated conspicuous acceleration in bone age and notably higher levels of sex hormones compared to the normal control group. The BMI of the ICPP group was significantly higher than that of the control group. Although the specific mechanism remains unclear, several recent studies have confirmed the reciprocal relationship between obesity and early female puberty (32–34). Therefore, emphasizing the screening of precocious puberty in obese children and enhancing weight management could be of significant importance for the prevention and treatment of early puberty. Additionally, the levels of IGF-1, insulin, HOMA-IR in the ICPP group differed significantly from those in the control group, consistent with previous conclusions (35–37).

Several studies have investigated the impact of changes in estrogen levels during the menstrual cycle on the ocular health of women, resulting in conflicting findings (20, 38). Another study suggested that elevated estrogen levels in women undergoing *in vitro* fertilization led to significantly increased ocular symptoms and tear film alterations (39). The study is the first study to examine the effects of increased estrogen on ocular parameters in girls with ICPP. Our findings indicate that the OSI in the ICPP group was significantly higher than that in the control group, but there were no statistically significant differences between other ocular parameters in the two groups. Furthermore, correlation analysis revealed a significant positive correlation between estrogen and OSI. OSI could serve as a novel auxiliary diagnostic method for dry eye (40), and we believe that these results at least suggest that estrogen might have a possible effect of estrogen on tear membrane stability in girls with ICPP. However, it is worth noting that estrogen is not a sensitive and specific laboratory parameter for the diagnosis of precocious puberty. Although our study showed a statistical correlation between estrogen and OSI, hormones that do not show significant correlation with ICPP should not be considered as important indicators, therefore, further research is required to explore the specific mechanisms underlying ocular parameter changes in ICPP. Additionally, further investigation is needed to determine whether peripheral precocious puberty also leads to similar ocular surface changes.

In addition to estrogen, other sex hormones may also affect the ocular surface of women (9, 41). Further exploration of the relationship with other hormones may be helpful to further understand the relationship between hormone levels and ocular surface parameters in ICPP girls. The impact of LH on the ocular surface has not been conclusively demonstrated. Studies have indicated that women with elevated LH levels in polycystic ovary syndrome exhibit greater ocular surface symptoms (42). However, in women undergoing *in vitro* fertilization, the coexistence of lower LH levels and more significant ocular surface symptoms and tear film changes has been observed (39). In this study, girls with ICPP showed slightly lower MNITBUT compared to the normal control group, although this difference was not statistically significant, consistent with findings in polycystic ovary syndrome women by Gonen et al. (42). Nevertheless, bivariate analysis revealed a significant negative correlation between LH and MNITBUT, suggesting might exist a potential influence of LH on NITBUT in ICPP girls, warranting further observation to clarify this correlation. The reasons for the inconsistent results between the different studies may be related to the different study populations involved, and variations in hormonal changes due to different physiological states, with the involvement of other sex hormones possibly affecting the ocular surface differently.

In this study, although the specific mechanism remains unclear, the impact of FSH on ocular surface parameters is consistent with that of LH. The protective effect of androgens on the ocular surface has been confirmed (11–13), but in this study, despite higher testosterone levels in girls with ICPP compared to the normal control group, no correlation between testosterone and ocular surface parameters was observed in the correlation analysis. The influence of progesterone on the ocular surface has received attention, but there is also a controversy (21). Elevated progesterone levels in late pregnancy (43, 44) and in women undergoing *in vitro* fertilization (39) suggest a possible association with aggravated dry eye symptoms and changes in ocular surface parameters. However, another study reported that physiological levels of serum progesterone during the menstrual cycle did not significantly affect tear production (45). In this study, no substantial changes in progesterone were observed in the ICPP group compared to the control group, and no significant alterations in ocular surface parameters associated with progesterone were observed.

In this study, although we conducted sample size calculations and deemed it sufficient to meet the expected scope and objectives of the research, we must acknowledge that the small sample size is indeed a significant limitation of this study. In future studies, we plan to increase the sample size to further validate and extend our findings. Secondly, the limited subjective expression of children was inherent in this age group, so the questionnaire was not included in the study. Due to the coordination problem of the participating children, we only did some examination of the ocular surface parameters, the Schirmer I tear test were not included in the study. Future studies with more ocular surface parameters are needed. Moreover, due to the differences in the pathogenesis between peripheral precocious puberty and ICPP, we only discussed ocular changes related to ICPP in the study. However, we believe that peripheral precocious puberty may also have a significant impact on ocular parameters, and this impact may differ from that of CPP. We did not investigate this aspect in the current study and will further elucidate it in future research. Nevertheless, our study is the first study to focus on sex hormone levels and ocular surface parameters in girls with ICPP. The current work provides a new perspective on ocular surface research and contributes to further mechanistic research related to ocular surface parameters in children with ICPP. In addition, our study takes into account the effects of gender differences on the ocular surface system (46, 47), Only girls with ICPP were included as the study object to avoid the influence of confounding factors.

In conclusion, when compared to the normal control group, girls with ICPP exhibited changes in the OSI. This variation may be linked to an increase in estrogen levels. Although there was a slight non-significant decrease in MNITBUT in ICPP girls, the negative correlation between LH and FSH with MNITBUT suggests avenues for further investigation. This finding is clinically helpful to pay attention to ocular surface parameters and have reasonable ophthalmic management in ICPP girls.

## Data availability statement

The raw data supporting the conclusions of this article will be made available by the authors, without undue reservation.

## Ethics statement

The studies involving humans were approved by the Ethics Committee of the Second Hospital of Shandong University. The studies were conducted in accordance with the local legislation and institutional requirements. Written informed consent for participation in this study was provided by the participants’ legal guardians/next of kin.

## Author contributions

WJ: Writing – review & editing, Writing – original draft, Formal analysis, Conceptualization. LY: Writing – review & editing, Writing – original draft, Investigation, Conceptualization. SL: Writing – review & editing, Writing – original draft, Investigation, Conceptualization.
